# HPV Positive Head and Neck Cancers: Molecular Pathogenesis and Evolving Treatment Strategies

**DOI:** 10.3390/cancers8040041

**Published:** 2016-03-29

**Authors:** Rüveyda Dok, Sandra Nuyts

**Affiliations:** 1 Laboratory of Experimental Radiotherapy, Department of Oncology, Katholieke Universiteit Leuven (KU Leuven), 3000 Leuven, Belgium; ruveyda.dok@kuleuven.be; 2 Department of Radiation Oncology, Leuven Cancer Institute, University Hospitals Leuven, 3000 Leuven, Belgium

**Keywords:** HPV, head and neck cancer, radiation therapy, molecular pathogenesis

## Abstract

Head and neck squamous cell carcinoma (HNSCC) is a highly heterogeneous disease that is the result of tobacco and/or alcohol abuse or infection with high-risk Human papillomaviruses. Despite the fact that HPV positive HNSCC cancers form a distinct clinical entity with better treatment outcome, all HNSCC are currently treated uniformly with the same treatment modality. At present, biologic basis of these different outcomes and their therapeutic influence are areas of intense investigation. In this review, we will summarize the molecular basis for this different outcome, novel treatment opportunities and possible biomarkers for HPV positive HNSCC. In particular, the focus will be on several molecular targeted strategies that can improve the chemoradiation response by influencing DNA repair mechanisms.

## 1. Epidemiology and Etiology of HNSCC

Head and neck cancers comprise a group of cancers that are anatomically located in the oral cavity, the oropharynx, the nasal cavity, paranasal sinuses, the nasopharynx, the hypopharynx and the larynx. Most of these (90%) cancers have squamous cell carcinoma histology and are called head and neck squamous cell carcinoma (HNSCC) [1,2].

HNSCC is classified as the seventh most common cancer worldwide with around 600,000 new diagnosis each year [3]. In the United States, 50,000 cases are diagnosed each year and nearly 10,000 deaths are attributable to this disease [4].

HNSCC develops mostly via one of the two primary carcinogenic routes, namely the chemical carcinogenesis through exposure to tobacco and alcohol abuse, which are known to be synergistic, and high-risk human papillomavirus (HPV) induced carcinogenesis [1,2,5,6,7]. Besides the exogenous risk factors certain inherited disorders such as Fanconi anemia show more susceptibility to HNSCC [2,8].

Interestingly, epidemiological studies demonstrated a decrease or stabilization of laryngeal, hypopharyngeal and oral cavity cancers. This decrease is ascribed to the gradual decrease of the use of primary exogenous risk factors (smoking and alcohol). In contrast, there is a clear increase in the incidence rates of oropharyngeal cancers mostly located at base of the tongue (BOT) and tonsillar region, which is ascribed to the increased incidence of HPV infections [9,10,11].

HPV related cancers (HPV+) are mostly located in the oropharynx (predominantly at the tonsils and tongue base), while only a small fraction of other HNSCC sub-sites have been associated with high-risk HPV infections [12,13,14]. Of note, there are discussions about the HPV infections associated with other HNSCC sub-sites with some seen as a non-specificity of the used HPV detection method [13,15,16]. A recent analysis performed by Chung *et al.* shows that HPV infections in other less common sub-sites are also clinically relevant [17]. A short overview with clinical and biological differences can be seen in Table 1.

The most common high-risk HPV types are HPV16, HPV18, HPV31, HPV33 and HPV35. These types are estimated to cause about 5% of the cancer burden worldwide, which includes 99% of cervical cancers, 25%–60% of head and neck cancers, 70% of vaginal cancers, 88% of anal cancers, 43% vulvar and 50% of penile cancers [18,19,20]. A significant subset of the 600,000 annual cases of HNSCC includes approximately 85,000 HPV associated (oropharyngeal) tumors, which means that the head and neck region is the second most common HPV+ tumor site. In 90% of the HPV associated tumors, HPV16 detection can be seen [18,20]. A noteworthy fact is that at current pace, oropharyngeal cancer incidence is expected to surpass cervical cancer incidence by 2020 in the United States [9,19,20].

The Centers for Disease Control and Prevention classifies high-risk HPV as the most common sexually transmitted infection in the United States and both oral and genital HPV transmission are associated with sexual activity. Interestingly, additional risk factors for HPV infections are tobacco, marijuana and alcohol use [13,21,22].

Apart from their different etiology and epidemiology, HPV+ HNSCC tumors show different patient and clinical characteristics, namely the patients tend to be younger at the time of diagnosis, less common tobacco and alcohol abusers and have a better socioeconomic status. Furthermore, HPV+ HNSCC are characterized by poorly differentiated or basaloid histology compared to the HPV− HNSCC with a generally keratinized histology. Moreover, the HPV+ tumors tend to have large nodal involvement and small tumor stage. As a consequence, the majority of HPV+ HNSCC patients are diagnosed at clinically advanced stages. However, they also tend to less likely develop secondary malignancies [10,13,23].

Interestingly, HPV+ HNSCC patients exhibit an improved outcome to the current treatment options compared to the HPV− HNSCC [24,25,26,27]. The biological basis for this improvement remains unclear. Several hypotheses have been proposed and will be discussed in the following paragraphs.

## 2. Molecular Pathogenesis of HPV Positive HNSCC

HPV are non-enveloped double-stranded circular DNA viruses with a genome of approximately 8000 kilo base pair There are more than 150 different types identified based on DNA sequence analysis and are divided evolutionary in genera but also divided according to their risk of cancer formation in high-risk and low-risk classes, with high-risk classes having a high association with cancer formation [28].

**Figure 1:**
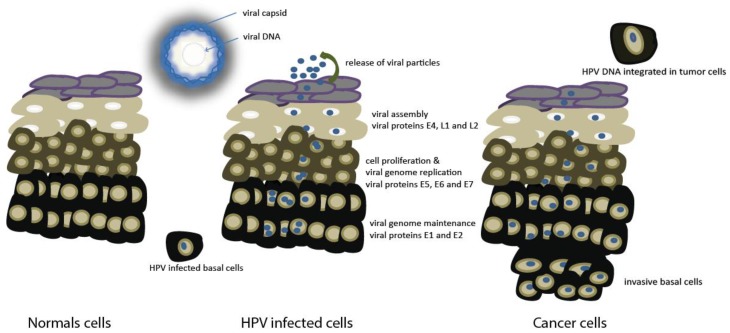
A short overview of HPV infection in cells.

The life cycle of HPV is a complex and tightly regulated process that makes use of the cell-cycle progression (Figure 1) [28,29].

Normal HPV infections are not associated with malignant transformation since the majority of HPV infections are spontaneously cleared and most patients show an effective immune response against subsequent HPV infections [13,21,30]. However, recent data show that high-risk HPV infections are cleared from oral cavity more slowly in comparison with low-risk HPV infections [30], suggesting the relation with the risk of cancer formation. Cancerous lesions are recognized by an increased risk of viral DNA integration into the host genome. This will result in destruction of E2 gene and higher expression of the oncogenes E6 and E7 in basal layer leading to a disruptive viral infection and incomplete viral life cycle and causes abrogation of cell cycle checkpoints [29].

E7 binds to cullin 2 ubiquitin ligase complex and results in the disruption and ubiquitination of pRB and other members of pRB pocket family [28,29]. Degradation of pRB protein by E7 will result in activation of E2F and subsequent transcription of S phase genes. The uncontrolled transcription of S phase genes also leads to the expression of p16INK4a (encoded by CDKN2A), a CDK inhibitor, as a negative feedback loop, which is also used as surrogate marker for HPV infections [28,29].

The function of E6 complements the function of E7. E6 binds to E3 ubiquitin ligases and results in degradation of TP53, which leads to cell cycle deregulation, due to loss of p21 function (CDK inhibitor) and loss of TP53 mediated apoptosis [28,29].

Although the HPV oncogenes stimulate cancer formation by promoting limitless replication potential and genomic instability, cancer formation is stimulated by secondary genetic events.

Before the knowledge of HPV infections and widespread use of (next generation) sequencing, HNSCC progression was also seen as an accumulation of stepwise (epi)-genetic alterations such as loss of chromosome 9p21 (CDKN2A loss), acquisition of TP53 mutations, 11q13 amplification (CCDN1), EGFR overexpression and PTEN inactivation [31].

Later on, HPV detection in the DNA of HNSCC patients resulted in a novel classification of head and neck cancers. It was clear that HNSCC are not only heterogeneous in means of their pathology and anatomical localization but also showed heterogeneity in regards to their biology. In 2012 three genetic subclasses were suggested by Leemans *et al.* being HNSCC cancer containing the transcriptionally active HPV that were classified as HPV+ tumors, HPV− tumors accompanied by high number of genetic changes (high chromosome instability (high CIN)), and HPV− tumors characterized by low CIN [2].

Recent progress in molecular technologies (the next generation sequencing and the omics era) gives definitely an in-depth picture of the molecular aberration in HNSCC. However, further classification of the heterogeneous group of HNSCC according to their predictive values (therapies) or even prognostic values is still in its infancy [6,32].

Nonetheless, it was shown that HPV+ tumors have less chromosomal copy number alterations compared to the HPV− HNSCC. The former is characterized by enrichment of 3q24-27 chromosomal amplifications, this region is coding for oncogene PIK3CA. The latter is characterized by gain of 11q13, a region encoding for cyclin D1 protein (encoded by CCDN1) [20]. Moreover, high throughput epigenetic screening experiments suggest differences in epigenetic profiles between HPV+ and HPV− HNSCC, with former characterized by hyper-methylated regions. However, further research is necessary to elucidate and validate these differences [33].

Also on expression level, several studies are performed and Chung *et al.* classified the HNSCC in 4 groups according to their expression profile: the classical, basal, mesenchymal and atypical group. The HPV+ group was classified in atypical group and was characterized by up-regulation of cell cycle and DNA replication genes [2,34]. Of note, this classification system was also used by The Cancer Genome Atlas (TCGA) database [35].

Initial mutational studies revealed that HNSCC have a relatively significant mutational overload with ranking 9th highest among tumors from 27 anatomical sites. These studies showed a 2 to 5 fold increase in mutation rates between HPV− and HPV+ HNSCC. However, the latter could not be verified by the recently published TCGA database, where the mutation rates between the two groups did not differ. It was noted that mutational profiles of HPV− HNSCC resembled the smoking-associated lung and esophageal SCC and was characterized with frequent transversions at CpG regions. The HPV+ HNSCCs meanwhile closely resembles the mutational profile of cervical cancers and showed higher mutation frequencies in PI3K pathway components and DNA repair genes [20,32,35].

## 3. Treatment Response

HNSCC treatment is based on combination of three major treatment arms, namely surgery, chemotherapy and radiotherapy (RT) [1,2]. For metastasized disease, generally systemic treatment like chemotherapy is preferred. However, in locally advanced disease, surgery and RT play an important role, with or without chemotherapy [1,36]. For early stage localized disease, RT and surgery seems to give similar results on locoregional control (LRC) and choice is made according to the organ preservation issue [2,37,38]. For locally advanced disease including HPV+ HNSCC, the addition of concurrent chemotherapy (platinum-based) to RT showed a five-year survival benefit of 6.5% and is often considered as standard care [39].

Interestingly, several retrospective and prospective trials have shown that HPV+ HNSCC patients have better overall survival (OS), disease free survival (DFS) and locoregional control (LRC) compared to the HPV− patients and this is independent of the treatment modality. In general the 5-year OS for HPV− HNSCC is around 50% while for HPV+ HNSCC patients values around 80% can be reached [13,24,26,27,40,41,42,43,44]. It should be highlighted that in these studies the HPV status is defined *post-hoc*. Of note, no data are available about the prognostic value of HPV on solely chemotherapy for primary HNSCC, since this is not the standard treatment modality. However, HPV status did show a correlation with outcome in metastatic and recurrent HNSCC patients treated with chemotherapy [45,46,47,48].

One of the landmark studies conducted by Ang *et al.* demonstrated a better 3-year OS (83.4% *vs.* 57.1%) and a 58% reduction of risk of death (HR = 0.4; 95% CI 0.27–0.66) in HPV+ HNSCC patients treated with (chemo)radiotherapy. What makes this study interesting is that the risk of death increased with each additional pack year of tobacco smoking. The authors suggested a novel classification system of HNSCC patients on basis of four factors: HPV status, pack-years of tobacco smoking, tumor stage and nodal stage [40]. The influence of smoking on the outcome of HPV+ HNSCC patient was recently verified by a pooled analysis of two randomized trials demonstrating that risk of death increases by 1% for each pack year of tobacco smoking or 2% for each year of smoking history of the patients [41]. One of the interesting observations about the performed studies involving RT is that HPV positivity results primarily in an improved LRC, but no statistically significant difference can be seen in distant metastasis rates [27,49].

These data suggest that the outcome of HPV+ HNSCC patients is strongly determined by radiation biology. Possible mechanisms influencing this response are highlighted in the following paragraphs.

## 4. Biological Basis for the Treatment Response

As previously mentioned, HPV+ HNSCC patients are associated with better outcome after treatment with RT [2,13,20,24,25,26,43,44]. The biological basis of this difference is still an intense area of investigation. 

Over the last couple of years several hypotheses have been put forward correlating RT response to micro-environmental (immune system and hypoxia) and tumor intrinsic factors [13,50]. It has been hypothesized that the immune system plays a more important role in clearance of HPV+ HNSCC compared to HPV− HNSCC due to the expression of viral proteins. Recent studies, showing an increase in immunogenic potential induced by RT, provide a solid base for the hypothesis in which RT outcome of HPV+ HNSCC is related to the increased immunogenic cell death induced by RT [13,51,52]. In concordance with this, preclinical data showed better tumor control after ionizing radiation (IR) in immune-competent HPV+ cell line based mouse models compared to immune-compromised mice [53].

One of the most studied environmental factors in relation to RT response is hypoxia, which is known to result in radiation resistance [6,13]. The most interesting study that highlighted the possible influence of hypoxia in radiation sensitization in HPV+ HNSCC was the retrospective sub-group analysis by the Danish Head and Neck Cancer (DAHANCA) group. This study showed that the hypoxic radiation sensitizer nimorazole did not improve the LRC in the HPV+ patient group [54]. However, currently published data regarding the association between HPV status and tumor hypoxia is ambiguous. Several studies showed no significant association between HPV and surrogate markers for hypoxia such as pO2 measurements, CAIX staining and hypoxic gene expression profiling [13,55,56,57,58]. However, a recently published study by Hanns *et al.* claimed that HPV related head and neck cancer showed lower expression of hypoxia related genes and which they relate to the ability to adapt to hypoxia [59]. However, it is possible to state that to date no clear experimental evidence is given for the resistance of HPV+ cancers to hypoxic sensitizers and the possible influence of hypoxia on RT sensitivity.

Several preclinical studies in HPV+ HNSCC indicated the importance of tumor intrinsic factors to the RT response. These studies can be classified in two categories according to the influenced pathways namely the influence of HPV on cell cycle and cell death pathways and the influence of HPV on DNA damage response (DDR) and DNA repair [13,60].

One of the first studies that not only showed the increased radiation sensitivity of HPV+ HNSCC cells but also investigated the possible influence of HPV on cell cycle progression and apoptosis was performed by Kimple *et al.* They demonstrated that expression levels of residual wild-type TP53 protein is enhanced by IR and resulted in prolonged G2/M phase arrest and cell death [61]. Interestingly, another study conducted by Pang *et al.* demonstrated that indeed introduction of E6 expression in HPV− HNSCC resulted in increased RT response by cell cycle regulation and cell death, but in a TP53 independent manner [62]. The accumulation of cells in G2/M phase with accompanied cell death and increased DNA damage was also described by Arenz *et al* [63].

Gubanova *et al.* showed that the expression of HPV oncogenes in HPV− cells results in promoter methylation and decreased expression of serine/threonine-protein kinase-1 (SMG-1), which resulted in radiation sensitization. Moreover, SMG-1 seems to correlate with HPV status and improved survival in HNSCC patients [64]. A second paper highlighted the importance of DNA repair in RT response. This study demonstrated that HPV+ cells have an impaired DNA repair, more specific defective double strand break (DSB) repair, and a prolonged G2/M phase after IR. No difference in apoptosis between HPV+ and HPV− cells was noted [65]. Interestingly, Park *et al.* ascribed the radiosensitivity of HPV+ cells to E7 oncogene induced delay in sub-lethal DNA damage repair [66]. In another study Dok *et al.* showed that HPV+ tumors have not only impaired DNA damage response, but also showed that this response was related to the expression of p16INK4A. Namely, they demonstrated that p16INK4A expression resulted in defective homologous recombination repair (HRR) by impairing the recruitment of RAD51 to the site of DNA damage. This function of p16INK4A was independent from its cell cycle regulatory function [67].

## 5. Biological Markers in HNSCC

An important and currently lacking aspect of targeted or personalized medicine is the stratification of patients who will benefit from novel treatment options or treatment adaptions, as it is currently tested in the de-intensification trials for HPV+ HNSCC patients. In this regard, the establishment of molecular markers is of utmost importance. The marker should be reliable, well-validated and easy to perform and interpret. The establishment of such robust therapeutic biomarkers has several challenges such as the heterogeneity of the tumors, role of clinical characteristics and additive influence of conventional risk factors such as tobacco and alcohol exposure [4,6,13].

Currently, HPV positivity in HNSCC cancers is accepted to be a prognostic marker for outcome and is currently assessed in several institutions. However, assessment of HPV infections has ongoing challenges with influence of classic risk factor such as tobacco exposure on the favorable outcome of HPV+ patients and the limitations in the diagnostic testing methods [6,13].

While HPV specific testing seems logical to use, the implementation in practice is complicated. Currently HPV testing can be divided into two categories, namely detecting the presence of the virus (direct methods) and using p16INK4a expression as biomarker for viral infections (indirect method) [13]. Viral DNA can directly be detected by southern blotting and by the highly sensitive Polymerase Chain Reaction (PCR). It should be noted that PCR techniques are known to have high false-positive rates due to their high sensitivity to HPV genome that may be present in tissue biopsies but which is unrelated to cancer [13,68].

This also highlights a major limitation of HPV-based DNA detection techniques namely these techniques detect the presence of HPV but not all HPV infections result in cancer formation. In other words, the clinically relevant HPV detection method is the one that is able to detect the transcriptionally active form of HPV [13,68].

There are several direct methods that correlate with biologically active HPV infections, including *in-situ* hybridization (ISH), RT-PCR for E6 and E7 mRNA and next-generation sequencing technologies. All of these techniques have the advantage that they have a high specificity and acceptable sensitivity, while the implementation in practice and the high costs are major disadvantages [13,68].

An alternative, which also detects transcriptionally active HPV, is the assessment of p16INK4a expression by IHC. P16INK4a expression correlates well with direct HPV detection methods as was shown in a pooled analysis comparing direct HPV detection methods with p16INK4a IHC with only in about 13% of cases discrepancies [36,68]. One of the major advantages of p16INK4a IHC is that it is a quick, inexpensive, and a readily available technique [13,68]. On top, several studies using p16INK4a IHC as a surrogate marker for HPV demonstrate that p16INK4a expression significantly correlates with outcome, independent of treatment modality. This is even the case after correction for other variables by multivariate survival analysis [44,69,70]. Moreover, recently these findings were verified in a meta-analysis [71].

However, one of the major disadvantages, that also prevent the general acceptance of p16INK4a IHC as gold standard, is the low specificity. The low specificity is systematically seen in several studies as 10%–20% of p16INK4a+ tumors are HPV−. Although several papers have shown the prognostic significance of p16INK4a even in the absence of HPV positivity there are contradictory studies showing significant poor survival rates for HPV−/p16INK4a+ HNSCC patients compared to HPV+/p16INK4a+ HNSCC patients [68,72,73,74,75].

The current debate around the specificity and correlation with outcome is the reason why several groups are cautious in using p16INK4a IHC as a standalone marker and are suggesting p16INK4a IHC as an initial screen for direct HPV detection methods. Of note, the cause of p16INK4a expression in HPV− cases is still unclear but likely to be due to mutations in p16INK4a/RB pathway [13,16,68,72]. Nevertheless, there is an emerging view that p16INK4a is a suitable single (surrogate) marker for HNSCC patient stratification but the universal guidelines for interpretation are lacking. One of the practical problems is the absence of a validated antibody. Second problem is interpretation of p16INK4a IHC, as currently the cut-off values vary from >10%–70% positive staining. Furthermore, p16INK4a expression shows differences in expression pattern, which makes the interpretation of the function of p16INK4a in these tumors difficult [13,16,68].

## 6. Possibilities to Increase the Current Therapeutic Window

The current treatment options are still suboptimal for both groups of HNSCC patients due to high resistance and recurrence (HPV−) and high toxicity (HPV− and HPV+) issues [2,38,60,76,77]. It should be kept in mind that the slopes of clinical dose-response curves indicate that enhancement of dose of RT by just 10% will increase tumor control rates by 5%–30% depending on tumor sites and current control rates [60,78]. Since it is not possible to increase the total radiation dose to the entire tumor due to high levels of normal tissue toxicity, novel therapeutic approaches are needed. The success of these novel treatments will be determined by understanding biological processes in HNSCC and several options are briefly mentioned in the following paragraphs [6,13,20,60].

### 6.1. De-Intensification of Current Therapy Options

Since HPV+ HNSCC patients have better therapy response rates, clinical trials assessing the possibility to de-intensify the current standard treatment options are ongoing [6,13,20]. These trials have reduction of acute and late toxicities associated with current aggressive treatment options in mind and can roughly be divided into two categories: de-intensification of chemotherapy by replacement by cetuximab, a chimeric monoclonal antibody against EGFR, and de-intensification of the radiation dose. Current trials de-escalating RT dose in HPV+ HNSCC patients are either in combination with induction chemotherapy or minimal invasive surgery. In these trials good responding patients are selected according to the response to the given neo-adjuvant treatment before a reduction in the radiation dose is made [13,79].

However, caution must be taken with this kind of trials since the significantly better response seen in HPV+ HNSCC patients can be a consequence of the received aggressive treatment. On top, development of de-escalation strategies can be detrimental in approximately 10% of HPV+ HNSCC patients with high risk of developing distant metastasis [49,80].

### 6.2. Targeted Molecular Agents

Although several laboratory studies show that targeting of aberrant oncogenic/mitogenic signal transduction pathways can result in radiation sensitization of tumors, translation of this combination treatment strategy to clinical trial settings is rare. Furthermore, it is noteworthy that multiple recent attempts to use molecular targeted agents for treatment of cancer patients have failed due to suboptimal dosing and scheduling as well as the lack of biomarkers that predict response to these targeted therapies [38,60].

Until recently, EGFR amplification or overexpression was seen as one of the most important aberrations in HNSCC patients leading to the development of EGFR inhibitors including monoclonal antibodies (mAb) as well as tyrosine kinase inhibitors (TKI) [4,6,20,32].

Cetuximab was one of the first developed and the only Food and Drug Agency (FDA) and European Medicines Agency (EMA) approved targeted agent for HNSCC patients in combination with radiotherapy in locally advanced disease or in combination with platinum-based chemotherapy in recurrent or metastatic disease [2,4,81]. However, the survival benefit (10%–15%) seen as a single treatment agent is disappointing. It is true that combination of cetuximab with radiotherapy showed significant improvement in LRC as well as OS without additional toxicity to patients [13,82]. However, because of the lack of clinical evidence for the superiority of this treatment over platinum-based standard therapies it is difficult to make straightforward conclusions regarding the value of this treatment option.

Additionally, genetic studies show that alterations in the EGFR pathway are rare (21%) and predominant in the HPV− population (15% HPV− *vs.* 6% HPV+), which suggest that these inhibitors are less likely to work in HPV+ HNSCC patients [20,32,35,83,84].

Interestingly, as mentioned before in the context of de-intensification of current treatment options for HPV+ HNSCC, three ongoing phase III trials (RTOG 1016, De-ESCALaTE and TROG 12.01) with similar concepts make use of cetuximab in combination with RT as a treatment arm and cisplatin in combination with RT as the standard arm [79]. The rationale for these studies is based on subgroup analysis from the Bonner trial testing the efficiency of cetuximab plus radiotherapy compared to radiotherapy alone [82,85]. Even though the study did not involve HPV testing, the patients who benefited most from concurrent cetuximab treatment had characteristics of HPV+ HNSCC patients. Recently, a secondary analysis of this study was performed in oropharyngeal cancer patients in whom the p16INK4a as the HPV status was determined retrospectively. The addition of cetuximab to RT increased the LRC, OS and progression free survival (PFS) in both patients with p16INK4a positive as p16INK4a negative head and neck cancers [86]. In line with these results the EXTREME trial showed that addition of cetuximab to the standard chemotherapy resulted in improved OS as well as PFS, both in p16INK4a/HPV+ and p16INK4a/HPV− HNSCC [45,48]. In contrast, the SPECTRUM trial, panitumumab (another EGFR monoclonal antibody) in combination with chemotherapy improved OS only in p16INK4a/HPV− patients [45,47].

Despite these discrepancies, there are still several EGFR inhibitors, mAb as well as TKI, in clinical trials. To increase the success rate of these kinds of therapies, the inhibition should not only be targeted but also biomarker driven [20,32,37,38,60].

Aberration and subsequent activation of PI3K pathway is one the most frequent events seen in HNSCC patients (34% in HPV− and 56% in HPV+) [35]. Activating mutations of the PIK3CA gene have been reported in 8%–21% of head and neck tumors, with an enrichment of mutations in HPV+ (37%) patients in comparison with HPV− patient population (18%) [20,32,35]. Furthermore, it has been reported that some HPV associated HNSCC cases showed only PIK3CA alterations. This suggests that PIK3CA mutations may have an important role in the development of HPV+ HNSCC as it has been hypothesized for cervical cancers [4].

Therefore, a tremendous interest has been shown for the inhibition of this pathway by mTOR/PIK3CA inhibitors. Although, the activity of PI3K inhibitors as single agents in lung squamous cell cancer patients with PTEN/PIK3CA mutations are disappointing. A preclinical study performed in HNSCC mouse model showed selective efficiency of PI3K inhibition in PIK3CA mutated samples [32,87,88]. Moreover, preclinical data indicate that PI3K pathways inhibitors show a great potential as radiation sensitizers. Based on these data currently a Phase Ib study, where the combination of pan PI3K inhibitor with weekly cisplatin and radiotherapy is tested, is ongoing in locally advanced HNSCC patients (NCT02113878). Additionally, several clinical trials are testing the combination of PI3K pathway inhibitors with chemotherapy or cetuximab [32,38].

**Figure 2:**
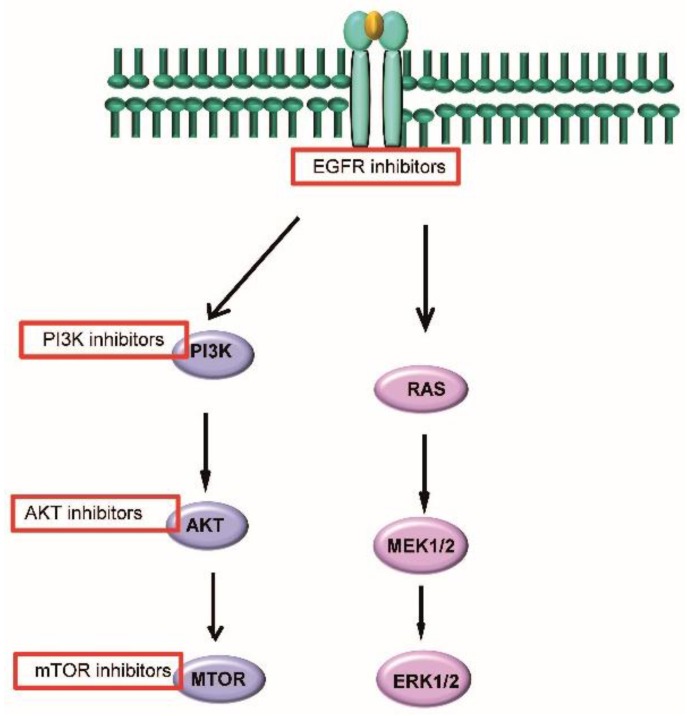
A short overview of targetable oncogenic pathways in HPV+ HNSCC.

In conclusion, although limited targetable oncogenic alterations show potential for the development of novel strategies for HPV+ HNSCC patients (see Figure 2 for a short overview). However, the importance and the current absence of stratification of patients to these strategies and the under-usage of RT combination strategies should be underlined.

### 6.3. Summary of DNA Damage Repair Mechanisms

RT or IR causes ionized molecules within biological tissues. These molecules are highly reactive and trigger a rapid cascade of damage affecting the molecules in cells. The high copy number of molecules or proteins will result in rapid turn-over, making the radiation induced damage to these molecules less significant for cellular survival. However, DNA has only two copies and a limited-turn over making DNA damage the most important mediator of cellular response to IR. Therefore the ability to sense DNA damage and control DNA repair is central for RT response [50,60].

As previously mentioned, effect of RT is directly linked to induced DNA damage which triggers DNA damage response (DDR). DDR on one hand initiates cell cycle arrest by checkpoint activation, giving the cell the opportunity to repair damaged DNA and on the other hand activates the DNA repair mechanisms. Especially double-strand breaks (DSB) have a high lethality when left unrepaired [50,60,89,90,91].

To understand the importance and possibilities of DNA repair, one should know the major DNA repair mechanisms in mammalian cells. The major repair mechanisms can be divided into five pathways. Most of the direct DSB are repaired by non-homologous end-joining (NHEJ), whereas replication associated DSB are repaired by HRR. Thus, the choice of repair is cell cycle dependent with NHEJ preferred in G0/G1 phase of cell cycle and HRR taking place during S/G2/M cell-cycle phases [89,90,91].

**Figure 3:**
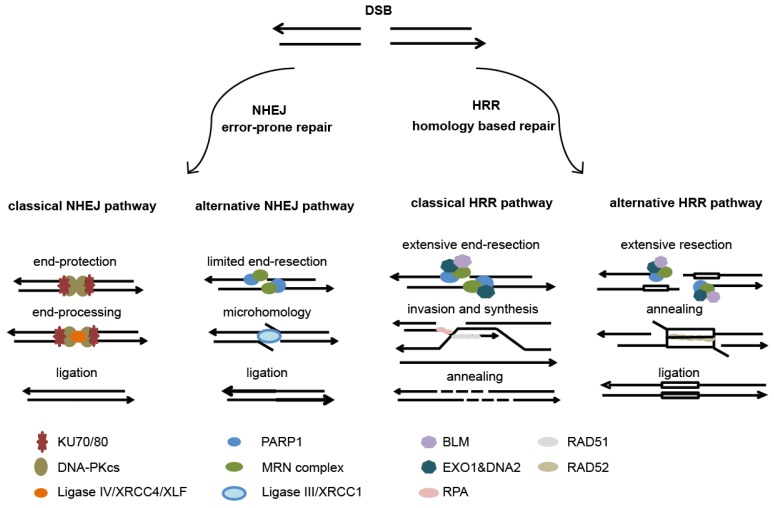
A short overview of DNA double strand break (DSB) repair mechanisms.

Characteristic for NHEJ is the lack of use of homologous sequences as repair templates, leading to an error-prone repair. The NHEJ is divided into two pathways being the classical pathway (c-NHEJ) and alternative pathway (alt-NHEJ) (see Figure 3 for a short overview).

The classical pathway directly ligates the free ends at DSB and is initiated by KU70/80 DNA end-binding. This binding protects broken ends and initiates the recruitment of the catalytic subunit or DNA-dependent protein kinase (DNA-PKcs) and the nucleases. The nucleases are necessary for free end-processing and will lead to more efficient ligation. Subsequent phosphorylation and activation of DNA-PKcs will result in the dissociation of the complexes from DNA-ends. This in turn will enhance the access of the Ligase IV/XRCC4/XLF protein complex, which completes the ligation reaction [89,90,91].

Defects in classic NHEJ proteins channels DSB toward the alternative NHEJ (alt-NHEJ) pathway. This pathway requires micro-homology and is regulated by PARP1, which will bind to the free DNA-ends instead of the Ku complexes. PARP1 binding will stimulate single-strand end-resection by the MRE11/RAD50/NBS1 (MRN) and CtIP protein complexes. Hereafter ligation will take place as is described for the classical NHEJ pathway. Since this pathway involves limited end-processing and micro-homology, it will result in even more inaccurate repair [89,90,91].

In contrast, HRR uses homologous DNA sequences as a template for reparation of DNA damage (see Figure 3 for a short overview). The genetic information is copied typically from sister chromatids in S/G2 phase and homologous chromosomes and repeated DNA sequences on chromatids can also be used. Because of the use of homology this mechanism is accepted as a more accurate way of DNA repair. The HRR process is also classified in an accurate and inaccurate pathway [89,90,91].

Both of the DNA repair processes start with single-strand DNA end-resection that is divided into two phases starting with limited end-resection initiated by MRN/CtIP complexes and is followed by extensive end-resection by helicases (BLM) and nucleases (EXO1 and DNA2) [89,90,91].

The accurate or the classical HRR pathway involves the binding of Replication protein A (RPA) to single-stranded DNA (ssDNA) that will result in stabilization of ssDNA which is necessary for binding and invasion of the homologous template strand, a process mediated by RAD51 nucleoprotein filaments. The invading strand is extended by newly synthesized DNA, which can subsequently anneal with the other resected-end. Finally, additional synthesis and ligation will result in high-fidelity repair. The inaccurate HHR or the single strand annealing (SSA) is characterized by the exposure of long complementary ssDNA repeats, which flank the DSB. Annealing of DNA is mediated by RAD52 leading to the deletion of one of the repeats and the DNA sequences between the repeats or to translocations when 2 DSB occur in different chromosomes [89,90,91].

The three other pathways operate on repair of single strand breaks (SSB) after induction with DNA damaging agents. These are termed base excision repair (BER), nucleotide excision repair (NER) and mismatch repair (MMR) and they use the complementary strand as a repair template [89,90,91].

It is becoming clear that these pathways do not act as separate entities and that there is a functional overlap between the pathways. This not only shows the complexity of DNA repair processes but also provides the opportunities to target these pathways to improve the radiotherapeutic index [60,89,91].

### 6.4. Targeting DNA Repair by Modulating Radiotherapy Response

Since RT results in DNA damage inhibition of DNA repair pathways can be exploited for radiation sensitization strategies. As mentioned before, deficiencies in DSB repair pathways are thought to be the most lethal lesions induced by IR. However, it has become apparent that acquired secondary DBS through deficient SSB repair is also important for the survival of cells after IR [60,89,91].

This knowledge led to the development of a range of novel compounds that influence DNA repair. For example inhibitors of important molecules in DSB repair, such as DNA-PKcs and PARP inhibitors have been shown to sensitize cancer cells to RT. Both strategies block DNA repair, thereby increasing damage in the treated cells and resulting in an increased cell death. Noteworthy is that such approaches do not necessarily provide selective eradication of cancer cells as they also influence normal cells [60,89,91].

Although cancer cells need repair mechanisms to survive they are also often defects in one or more aspects of DNA repair, which lead to an addiction and reliance to the other/back-up DNA repair pathways. This overreliance or addiction of cancer cells for specific DNA repair pathways can be therapeutically exploited by inhibiting the back-up DNA repair pathways and is called synthetic lethality. The best example of a synthetic lethality approach is the use of Poly (ADP ribose) polymerase (PARP1) in BRCA1/2 mutated breast and ovarian cancers. The inhibition of PARP1 in these cancers resulted in the accumulation of single strand breaks (SSB) leading to DSB and eventually cell death upon cellular replication [60,89,91,92].

In line with this, cancer cells with aberrations in their repair mechanisms are expected to shift their repair to less common used back-up DNA repair pathways in response to IR, this compared to normal cells where the common used DNA repair mechanism is still intact. Inhibition of the back-up DNA repair pathway used by the tumor cells can result in a relative tumor selective radiation sensitization [60,91].

Another advantage of combining synthetic lethal drugs with DNA damaging agents, like RT instead of using as a single therapy modality, is the possibility to avoid resistance. Since synthetic lethal drugs inhibit DNA repair components, it will also result in an increased mutation rate of the repair pathways and as a consequence the cancer cell can activate the repressed DNA repair pathways by additional mutations as it is noted for BRCA2 and PARP inhibition [60,91,93].

## 7. Conclusions

It is clear that HPV related HNSCC form a distinct entity and that current treatment options are not answering the need of these patients. De-intensification of current therapeutic schemes with molecular targeted agents in combination with standard treatment forms an interesting strategy to increase the (radio)-therapeutic index. However, these strategies also highlight the importance and need for prognostic and predictive biomarkers for stratification of patients. Emerging data generated by high-throughput technologies will give us valuable information in this regard but it will also bring novel challenges regarding to interpretation of clinical relevance of these data and feasibility to clinical translation. This highlights the importance of molecular validation of biological data, but also indicates the importance of understanding and anticipating the possible interaction between different treatments.

## Figures and Tables

**Table 1:** Overview of clinical and biological differences between HPV positive and HPV negative head and neck cancer patients.

	HPV Positive	HPV Negative
**Clinical, epidemiological characteristics**		
**Incidence**	Increasing	Decreasing
**Age**	Younger	Older
**Socioeconomic status**	Higher	Lower
**Risk factors**	Sexual behavior, marijuana exposure	Tobacco and alcohol exposure
**Location of the tumor**	Oropharynx (common in tonsil and BOT)	All head and neck sites (common in floor of mouth, lateral tongue and ventral tongue)
**Prognosis**	good	poor
**Biological and histopathology characteristics**		
**TP53 pathway**	E6 mediated degradation	TP53 mutations
**RB pathway**	E7 mediated degradation	Inactivating mutations or other alterations in pathway
**p16INK4a expression**	Commonly overexpressed	Commonly decreased expression (inactivating mutations and hyper methylation)
**Histology**	Poorly differentiated or basaloid SCC	Modestly to well differentiated, keratinized SCC

Abbreviations: HPV, Human papillomavirus; BOT, Base of tongue; SCC, squamous cell carcinoma.
